# Social Media Dimensions and Productivity Among Healthcare Workers: Evidence from a Nigerian Tertiary Hospital

**DOI:** 10.3390/healthcare13151836

**Published:** 2025-07-28

**Authors:** Precious Chisom Uzoeghelu, Mary Agoyi

**Affiliations:** 1 Department of Management Information Systems, Cyprus International University, Haspolat, Nicosia 99258, Turkey; 2 Department of Information Technology, Cyprus International University, Haspolat, Nicosia 99258, Turkey; magoyi@ciu.edu.tr

**Keywords:** social media, Social Networking Site, health information, healthcare, professional engagement, productivity, information, communication

## Abstract

**Background**: Social media platforms play a crucial role in contemporary healthcare, facilitating patient participation and enabling communication among healthcare workers, as well as serving as a platform for medical awareness and advocacy. Social media use among healthcare workers has increased to 91%, with 65% using it for health promotion purposes. Nonetheless, current studies have not properly and empirically explored its dimensions. **Objectives**: This study therefore examines social media dimensions and the productivity of healthcare workers. **Methods**: Leveraging the professional productivity theory and digital engagement theory, the study employs SPSS to analyze the gathered data through a partial least squares (PLS-SEM) approach to explore social media dimensions and productivity among healthcare workers in a Nigerian Tertiary Hospital. Based on a cross-sectional descriptive survey design and stratified random sampling method, 344 medical workers were analyzed. **Findings**: The study found that fear of missing out, information sharing, social influence, trust, and social media usage have a significant impact on the productivity of healthcare professionals. **Conclusions**: This research adds to the growing academic research on the capabilities of social media within the circular economic systems aimed at advancing healthcare delivery in developing economies. The research offers a method for maximizing the use of social media within healthcare settings to foster enhanced healthcare outcomes, particularly productivity.

## 1. Introduction

Social media has revolutionized the way healthcare professionals communicate, collaborate, and share information. In 2024, about 4.89 billion people use social media platforms effectively, while 5.18 billion people worldwide access the internet, accounting for more than 65% of the global population [1]. This rapid digital integration has extended into professional domains, particularly among healthcare workers, where social media is used to exchange clinical information, engage in professional development, and foster collaborative patient care [2]. The utilization of social media in healthcare settings has grown significantly, creating a multifaceted impact on productivity across various contexts. Furthermore, social media platforms such as Twitter, LinkedIn, and Facebook function as online networks through which healthcare workers participate in evidence-based discussions and interact with colleagues globally. This facilitates information sharing that leads to better patient care [3]. Patients are actively involved with social media, utilizing it for health education, peer networks, and communicating with healthcare workers. This transition to patient-oriented care promotes honest interaction and collaborative decision-making and strengthens trust between patients and providers. Consequently, healthcare workers are adjusting to these emerging standards by establishing an active virtual presence and utilizing social platforms to enhance participation and medical delivery [4]. The core of the study examines how significant social media-linked factors, fear of missing out (FoMO), information sharing, social influence, and trust influence the productivity of healthcare workers [5].

Productivity in this setting is conceptually defined as the capacity of healthcare workers to perform their work efficiently, uphold career engagement, and improve patient healthcare through the competent use of online tools. Theoretically, within the context of healthcare professionals, productivity is defined as the ability and proficiency with which individuals accomplish their occupational roles, which include medical care, managerial responsibilities, career advancement, and patient engagement. Productivity exceeds conventional measures of work results, which include technological skills, cooperative participation, and knowledge adaptability facilitated by social media. This theoretical foundation is supported by two fundamental theories: Digital Engagement Theory (DET), which proposes that continuous, intentional engagement with online platforms such as social media can encourage more profound participation, improve intellectual focus, and develop professional resources within connections. Based on this theory, healthcare workers who are active on professional online networks exhibit increased drive, responsiveness, and collaborative behavior. These factors improve production as well as contentment. PPT proposes that productivity in a professional environment is an evolving outcome of both personal skills and organizational culture. Social media functions as an essential facilitator by encouraging information sharing and non-centralized collaboration, which are vital for contemporary healthcare delivery. Together, these theories define productivity not merely as a fixed outcome but as a dynamic system influenced by information sharing, reducing FoMO, social media influence, and productivity. Each of these is facilitated by social media platforms.

Thus, the theoretical definition of productivity in this study reflects a holistic construct involving task efficiency, professional engagement, knowledge sharing, adaptability, and enhanced service quality, all within the framework of digital interaction. Therefore, the theoretical explanation in this research demonstrates an integrated model that includes information sharing, reduced FoMO, social media influence, and productivity, all structured around social media usage.

Although the concept of a research gap is recognized, previous research has mainly highlighted the adverse effects of social media in medical settings, which include the dissemination of false data, patient privacy issues such as the spread of misinformation, breaches of patient confidentiality, digital exhaustion, and job-related burnout [6]. However, these research works commonly ignore the detailed and possibly beneficial components of social media usage, especially its impact on work productivity. This study diverges from previous publications by refocusing from the broad threats of social media to the possibility of productivity among healthcare workers.

By refining the scope to these key elements, the research seeks to offer an in-depth understanding of how social media operates as an effective resource in enhancing healthcare delivery and advancing results among healthcare workers. This research consequently explores dimensions of social media and productivity among healthcare workers by assessing how it assists them to enhance productivity, job quality, and execute their responsibilities efficiently [7]. It further strives to address existing gaps in the literature by highlighting current patterns, specifically concerning FoMO, trust, information exchange, and social influence within healthcare settings. This approach aims to offer a well-defined rationale and a stronger consistency between research objectives and wider research developments.

## 2. Theoretical Foundations and Hypothesis

This research is based on two essential theoretical models, which include Professional Productivity Theory and Digital Engagement Theory. This collectively explains social media dimensions and productivity among healthcare workers. Professional Productivity Theory asserts that productivity in a professional setting is optimized by combining the motivational, environmental, and social dynamics, such as well-defined goals, acknowledgment of accomplishments, supportive work environments, and clear communication. Within the context of this research, this framework proposes that productivity can be improved when social media is used to promote cooperation and information sharing, trusted by healthcare workers. Nevertheless, productivity can be hindered when social media becomes a cause for distraction. This model clarifies ways social media can regulate productivity among healthcare workers. Digital Engagement Theory states that social media has positive and negative effects on healthcare professionals. The Digital Engagement Theory can be connected to the study on social media dimensions and productivity among healthcare workers by analyzing how digital engagement impacts the work performance of healthcare workers. Social media offers access to pertinent healthcare information and colleague discussions among healthcare workers. As a result of social influence and fear of missing out on information sharing, healthcare workers can be negatively influenced, disrupting productivity. Collectively, these models establish a framework for analyzing the intricate relationship between social media usage and productivity among healthcare workers. The variables explored in this study—FoMO, information sharing, social influence, trust in the system, and social media usage—are based on these theoretical frameworks. The integration of Professional Productivity Theory and Digital Engagement Theory presents a thorough foundation for elucidating work productivity within digital workspaces. Through the combination of these theories, scholars can examine the role of digitalization on productivity and how the dynamics of engagement influence work success. These theories can provide knowledge on the impact of fear of missing out, social influence, information sharing, and trusting the system on productivity and professional engagement.

### 2.1. Fear of Missing out on Social Media Usage

FoMO is the continuous anxiety that one is excluded from valuable situations that their colleagues are experiencing. In this digital age, FoMO is increased by uninterrupted connectivity and the circulation of knowledge on social media applications. For healthcare workers, FoMO can trigger excessive monitoring of notifications and posts, interfering with work operations and attention. However, when regulated, this conduct can also notify them of the current advancements, guidelines, and medical information. According to the Digital Engagement Theory, FoMO signifies a psychological stimulus that affects digital activity and can either strengthen or weaken engagement standards. According to DET FoMO is a trigger of online behavior, possibly enhancing participation. In professional settings, however, FoMO may result in habitual reviewing, which disrupts concentration and scheduling, which is a component of PPT. Therefore, FoMO can interrupt healthcare workers and influence productivity in multifaceted ways. Thus, the following is hypothesized.

**Hypothesis** **1** **(H1):** *Fear of missing out on social media usage has a significant impact on the productivity of healthcare professionals.*

### 2.2. Trust of the System on Social Media Usage

Trust is the degree of belief individuals have in the consistency, protection, and usefulness of social media networks. Trust denotes the extent of certainty healthcare workers have in the honesty, safety, and consistency of digital networks, encompassing social media and communication tools. In the healthcare setting, where privacy, precision, and prompt availability of content are essential, a dependable platform alleviates professional doubt and encourages regular utilization of fundamental and confidential activities. These may include patient online discussions with associates, disseminating medical interpretations, contributing to medical education, or obtaining healthcare information. If people perceive social media platforms as safe and trustworthy, they are inclined to utilize them for professional activities. According to the Digital Engagement Theory, trust is crucial for continuous and significant digital engagement among healthcare workers. The Professional Productivity Theory acknowledges the role of functional and reliable resources in supporting job productivity and minimizing reluctance to embrace technologies. Thus, the following is hypothesized.

**Hypothesis** **2** **(H2):** *Trust in the system in social media usage has a significant impact on the productivity of healthcare professionals.*

### 2.3. Social Influence on Social Media Usage

Social influence is defined as the impact colleagues, advisors, and professional contemporaries have on a person’s acceptance and utilization of social media. Originally, in the healthcare context, observing others benefit from professional social media use may motivate relatively productive conduct. As stated by the Digital Engagement Theory, social influence leverages perceptions regarding technology use as well as enhances the recognized worth of involvement. PPT proposes that such influences can improve productivity by encouraging the adoption of more productive practices. Thus, the following is hypothesized.

**Hypothesis** **3** **(H3):** *Social influence in social media usage has a significant impact on the productivity of healthcare professionals.*

### 2.4. Information Sharing on Social Media Usage

Social media helps in gaining new ideas, skills, or techniques, and it helps healthcare workers avoid situations that can expose them to contracting diseases [8]. Information sharing includes the exchange of concepts, procedures, and developments across professional platforms through social media networks. For healthcare workers, this consists of medical insights, study findings, and their colleagues’ perspectives. Based on the Professional Productivity Theory, the availability of current and appropriate information positively enhances decision-making and work productivity. The Digital Engagement Theory also validates that information sharing on social media improves professional networks and expertise growth. By being mindful of these factors, individuals and organizations can effectively utilize social media for information sharing [9]. Thus, the following is hypothesized.

**Hypothesis** **4** **(H4):** *Information sharing on social media usage has a significant impact on the productivity of healthcare professionals.*

### 2.5. Social Media Usage on Productivity

Due to the excessive use of social media, its benefits and disadvantages are multifaceted. It can be used for information dissemination as well as pose a distraction in the workplace. This could lead to a failed task and procrastination among healthcare workers. This study shows that the proper balance between social media usage and professional engagement can lead to efficient productivity. Thus, the following is hypothesized.

**Hypothesis** **5.** **(H5).** *Social media usage has a significant impact on the productivity of healthcare professionals*.

## 3. Research Methodology

### 3.1. Measurement Scales

The items measuring the construct in the model were adopted from existing literature and measured by using a five-point Likert scale with an endpoint of 1 as strongly disagree and 5 as strongly agree. The measurement items are displayed in Appendix A.

#### 3.1.1. Study Design

This research employed a quantitative cross-sectional exploratory research design to explore social media dimensions and productivity among healthcare workers. The design was selected to obtain an overview of the present practices and viewpoints among the target population during a specified period.

#### 3.1.2. Population and Sampling Technique

The target population consists of healthcare workers at Nnamdi Azikiwe University Teaching Hospital (NAUTH), including doctors, nurses, pharmacists, laboratory technologists, midwives, ward supervisors, and ward clerks. To guarantee a sufficient inclusion of all professional groups, stratified random sampling was employed. The population was first separated into seven similar strata according to the job functions. For each of the strata, simple random sampling was employed to select the respondents. This guaranteed that every qualified participant possessed a fair opportunity for inclusion within their professional category, enhancing representativeness and minimizing sampling distortion. Inclusion criteria consisted of employed staff who are working presently and who utilize different social media platforms. Exclusion criteria consisted of trainees, contract employees, and individuals reluctant or incapable of offering informed consent.

#### 3.1.3. Sample Size

The sample size was determined using Cochran’s formula for sample size calculation, which is suitable for extensive demographics. We found a 95% confidence level (Z = 1.96), a margin of error of 5% (e = 0.05), and a response distribution of (*p* = 0.5). The initial necessary sample size was 384. Nevertheless, considering possible restrictions in accessing the respondents, a reduced but valid sample size was obtained. Eventually, 344 responses were gathered. This guaranteed the statistical reliability of the research.

#### 3.1.4. Instrument for Data Collection

A structured questionnaire was designed based on a previously conducted empirical study, modified in relation to the healthcare context. The survey comprised measuring variables like fear of missing out (FoMO), social media trust, social influence, information sharing, social media usage, and productivity. To guarantee content validity, a pilot test was conducted, and about 20 healthcare professionals who were not part of the study helped improve vague questions and unclear language. Also, expert evaluations were integrated.

#### 3.1.5. Validation and Reliability

Construct validity was performed using average variance extracted and composite reliability. Measurement reliability was guaranteed using Cronbach’s alpha to evaluate internal consistency.

#### 3.1.6. Data Analysis

Data was examined using Partial Least Squares Structural Equation Modeling (PLS-SEM) via SmartPLS 4. This method was adopted because of its adequacy for predictive modeling, managing intricate models including several variables, and because of its strength. Multicollinearity was examined using the Variance Inflation Factor (VIF). Normality was not precisely needed due to the non-parametric characteristic of PLS-SEM, but skewness and kurtosis were examined for significant anomalies.

#### 3.1.7. Ethical Considerations

Ethical approval was obtained from the Ethics Committee of Nnamdi Azikiwe University Teaching Hospital with the approval code NAUTH/CS/66/VOL.17/VER.3/127/2024/130 on 16 January 2025. Informed consent was obtained from all participants. The study ensured anonymity and confidentiality, and participants were asked to respond freely.

### 3.2. Common Method Bias

The common method bias was assessed through the inner model’s Variance Inflation Factor (VIF) values. In the current study, all the VIF values are lower than 3.33, so the model can be considered free from common method bias [10].

### 3.3. Structural Model Analysis

**Figure 1:**
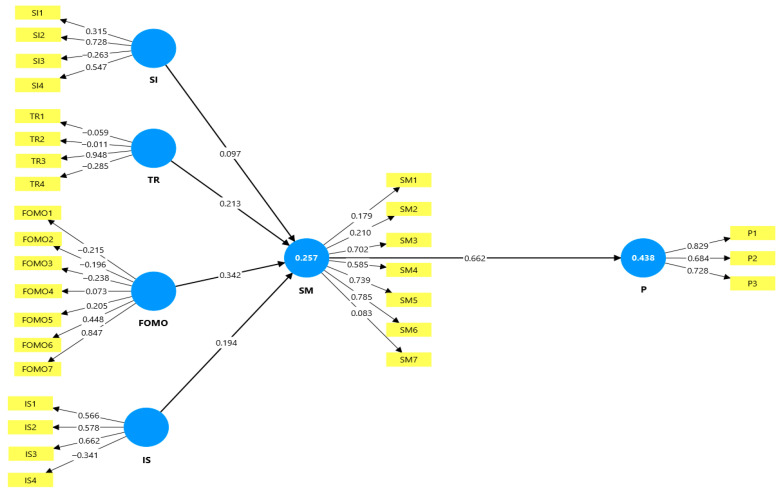
Path analysis.

Figure 1 shows the findings from testing the different assumptions. To do this, the study considered the structural model’s *p*-values, the original coefficients (β), and the significance of the independent and dependent constructs. The different correlations between the adopted constructs are shown in Figure 1. As can be seen, all the constructs had a positive association with one another. To further examine the different links, hypothesis testing was used. 

## 4. Result

The Statistical Package for Social Science (SPSS version 27) was used to analyze the gathered data, which was then displayed in frequency and percentage tables to describe the characteristics of the sample. The study also employed SmartPLS 4 to perform partial least squares structural equation modeling to test the hypothesis. The study used the PLS-SEM technique because PLS is best suited for exploratory studies with new variables [11]. Thus, the data in this study were regarded as appropriate for the factor analysis to proceed.

Table 1 displays the results of the measurement model assessment, including factor loadings, Cronbach’s alpha (α), composite reliability, and average variance extracted (AVE) for all constructs: social influence (SI), trust (T), fear of missing out (FoMO), information sharing (IS), social media usage (SMU), and perceived productivity impact (PI).

Table 2 shows the Fornell–Larcker criterion values used to assess discriminant validity among the constructs. The diagonal elements represent the square roots of the average variance extracted (AVE), while the off-diagonal elements are the inter-construct correlations.

Table 3 summarizes the hypothesis testing results for the direct effects between constructs. All hypothesized paths (H1–H5) were found to be statistically significant, with *p*-values less than 0.05, indicating that all proposed relationships are supported by the data.

Figure 1 shows the structural model output from the PLS-SEM analysis, including the path coefficients, outer loadings, and coefficient of determination (R^2^) values for the endogenous constructs social media usage (SM) and productivity (P).

Table 4 presents the structural model results, including path coefficients (β), *p*-values, and effect sizes (f^2^) for the hypothesized relationships (H1–H5). All five hypotheses were supported, as indicated by statistically significant *p*-values (*p* < 0.05).

Table 5 presents the coefficient of determination (R^2^) values for the two endogenous constructs: social media usage (0.663) and productivity (0.723). These R^2^ values indicate the proportion of variance explained by the predictor variables in the structural model.

Table 6 reports the Q^2^ (Stone–Geisser’s Q^2^) values obtained to assess the predictive relevance of the model for the endogenous constructs social media usage (0.024) and productivity (0.129). A Q^2^ value greater than 0 indicates predictive relevance.

## 5. Discussion

### 5.1. Interpretation

The reliability outputs of the measured items are displayed in Table 1. Cronbach’s alpha is one of the most used reliability coefficients, which is used to check the internal consistency of a set of items or questions to measure a given variable. Cronbach’s alpha scores move between 0 and 1, and greater scores indicate greater reliability. Reliability analysis is key to confirming the quality of the data gathered and the credibility of the conclusion. The researcher can thus infer if the measurement instrument measures what is truly being studied in the variables without significant errors or inconsistencies. Cronbach’s alpha is employed to evaluate the reliability of the variables in this study. It presents the measurement of the internal consistency for social influence, trust in the system, fear of missing out, information sharing, social media usage, and productivity. Prior to assessing the path model, the measurement model was analyzed to ascertain the reliability and validity of the latent variables. Cronbach’s alpha (α) was used to evaluate the internal reliability of each variable, where values range between 0 and 1, with higher values indicating a stronger reliability. A threshold of 0.70 is recommended for validated scales. Constructs like social influence (α = 0.614), trust in the system (α = 0.592), and fear of missing out (FoMO) (α = 0.584) had a threshold below 0.70. However, in exploratory research, values lower than 0.70 can still be acceptable, particularly when dealing with newly modified scales or variables being applied in unique contexts [10]. Although the alpha values were slightly low, the composite reliability (CR) for these variables ranged from 0.556 to 0.826, and their average variance extracted (AVE) results surpassed the minimum threshold of 0.50, supporting convergent validity. This suggests that, although internal consistency was average, the constructions accounted for an adequate amount of variance in their observed item.

The integration of key statistical indicators improves the robustness of the measurement model. The Standardized Root Mean Square Residual (SRMR) was recorded as 0.066, which is below the accepted threshold of 0.08; this indicates a good model fit. Also, the coefficient of determination (R^2^) values was 0.663 for Social Media Usage (SMU) and 0.723 for Productivity (P). This suggests that 66.3% and 72.3% of the variance in these endogenous variables, respectively, is explained by the model. These R^2^ values demonstrate substantial explanatory power. Convergent validity focuses on the extent to which measures of a given construct are highly correlated, suggesting that they are equally measuring the same factor. This is usually measured using the average variance extracted (AVE); any figure higher than 0.5 is usually regarded as an indicator of good convergent validity. As seen in Table 1, social influence (SI), trust (T), fear of missing out (FoMO), information sharing, social media usage, and productivity (P) displayed good convergent validity with figures above the threshold.

Composite reliability (CR), which measures internal consistency, showed mixed findings. A value above 0.70 is generally acceptable, although values between 0.60 and 0.70 are acceptable for exploratory research. These CR values were identified as follows: SI = 0.288, T = 0.557, and *p* = 0.693. Productivity, which was at 0.672, was below the benchmark, but still acceptable according to exploratory research. Trust and social influence fell below the threshold, with CR values of 0.557 and 0.288, respectively. Although both constructs were retained based on theoretical justification and precedents in prior literature, findings related to these constructs should be interpreted with caution due to concerns regarding their internal consistency, since this study is exploratory [12].

Discriminant validity was examined using the Fornell–Larcker criterion. This states that a construct must correlate more with its indicators than with other constructs. As shown in Table 2, the square root of the AVE (diagonal values) exceeds the inter-construct correlations (off-diagonal values) in most cases. For instance, FoMO (0.813), IS (0.864), P (0.825), SI (0.872), SMU (0.790), and T (0.833) all satisfy this criterion, confirming discriminant validity.

Despite some constructs falling below the accepted threshold of 0.5, constructs with loadings as low as 0.20 were retained. This was justified based on the sample size of 344. Samples above 300 with factor loadings as low as 0.2 can be considered statistically significant. This is because of the low loading values in the large sample size, and the construct was also needed for the study [13].

It is also important to acknowledge that the single-center design of this study limits the generalizability of the findings. Hence, the findings should be interpreted within the framework of the study setting, and caution should be taken when applying these findings to broader populations.

### 5.2. Theoretical Explanation

These results are in line with the Digital Engagement Theory, which asserts that enhanced utilization of digital tools promotes professional cooperation and productivity. They also conform to the Professional Productivity Theory, which underlines the role of immediate and appropriate information in increasing personal and organizational productivity. The integration of model fit indices, R^2^ values, and construct validity measures provides a thorough verification of the measurement model and demonstrates the theoretical relevance and functional utility of the variables used in this research. The R^2^ values of 0.663 and 0.723 obtained in this research show that about 66.3% and 72.3% of the variance in the corresponding dependent variables is justified by the model. R^2^ values above 0.60 indicate a significant explanatory power, specifically in healthcare research where human actions, perceptions, and institutional factors often introduce complications and variability [14].

In exploratory studies involving Partial Least Squares Structural Equation Modeling (PLS-SEM), such R^2^ values are considered strong and adequate to validate the predictive accuracy and theoretical soundness of the model [15]. The ability of the model to explain more than two-thirds of the variance in the outcome variables, such as social media usage and productivity, suggests that the proposed theoretical framework, grounded in the Digital Engagement Theory (DET) and the Professional Productivity Theory (PPT), accurately explains the key determinants affecting these results.

### 5.3. Comparative Studies

In addition, similar studies on technology adoption, digital participation, and healthcare workforce actions have reported comparable R^2^ values, strengthening the validity and reliability of these results [16,17]. As such, these R^2^ values provide strong empirical justification for the model’s adequacy in explaining the targeted productivity. The obtained Q^2^ values of 0.024 and 0.129 suggest that the model demonstrates limited to moderate predictive ability, in line with the benchmark criteria [18,19]. Specifically, a Q^2^ value greater than 0 confirms that the model possesses predictive strength, while values surpassing 0.09 are representative of moderate forecasting capacity. Additionally, incorporating behavioral factors such as social influence, trust, and fear of missing out (FoMO) naturally introduces variability, making exceptionally high Q^2^ values relatively rare. Therefore, the values recorded are consistent with established research benchmarks for models utilizing Partial Least Squares Structural Equation Modeling (PLS-SEM) within healthcare and technology-related studies [20]. Therefore, these Q^2^ results deliver sufficient substantiation for the model’s forecasting reliability concerning the main dependent factors explored in this research.

#### Summary of Hypothesis Test Results

This research analyzed how social media dimensions influence social influence, trust in the system, fear of missing out (FoMO), information sharing, and productivity, thereby affecting healthcare workers’ participation in online platforms. Guided by the Digital Engagement Theory (DET) and Professional Productivity Theory (PPT), the results offer a detailed comprehension of how online behaviors and professional results are connected in intense healthcare contexts. The research contains explanatory notes and hypothesis-testing portions, which aim to validate the variables’ relationships. The described assumptions receive accounting validation through statistical examinations in this section, even though the relationships remain generalized. Statistics reveal the connections between the identified constructs of perceived usefulness and customer satisfaction, the intention to continue, and many more. Behind the results of hypothesis testing, researchers find both confirmations of their theoretical structures and observations regarding how respondents make behavioral choices. This section is the core component for establishing conclusions from analysis results that contribute to resolving the research objectives and questions.

**H1:**  *Fear of Missing Out (FoMO) → Social Media Usage.*

The first hypothesis was significant (β = 0.430, *p* = 0.000), indicating a strong and positive link between social media usage and FoMO. This finding is in line with the Digital Engagement Theory, which states that progressive online engagement facilitates reliance and fear of missing critical updates [21]. Healthcare workers experience FoMO in different ways, such as fear of missing new research findings, policy changes, or colleague interactions, hence promoting social media use to enhance productivity. The first hypothesis (H3: social media usage → fear of missing out) is also significant and indicates a positive and strong relationship (β = 0.519, *p* = 0.000), such that social media users tend to have FOMO. Alignment with previous findings suggests that people who are afraid of missing critical updates or professional conversations utilize social media platforms to stay informed [21]. The healthcare sector demands frequent social media engagement, considering that new medical advancements, guidelines, and trends are emerging. FOMO plays the role of a motivator quite effectively.

**H2:**  *Trust → Social Media Usage.*

The second hypothesis was assessed (β = 0.416, *p* = 0.003), suggesting that trust in social media platforms enhances adequate social media usage. According to the Digital Engagement Theory, trust is a core variable that facilitates individuals to engage with and depend on social media use. Trust increases when healthcare workers view online platforms as safe, confidential, and dependable [22]. This positive relationship is supported by an empirical study that points to the acceptance of online tools in healthcare being credible [23]. Secondly, hypothesis 2 (H2: trust → social media usage) is accepted at 0.416, a significance of 0.003, suggesting a positive relationship between trust and social media usage. Trust in digital platforms further increases users’ willingness to adopt and participate actively in social media-based professional interactions [22]. As such, studies have shown how medical personnel use social media, and it has been found that security, data privacy, and credibility issues affect how social media is used for professional purposes. Therefore, trust in social media systems can lead to great adoption rates in healthcare settings.

**H3:**  *Social Influence → Social Media Usage.*

The third hypothesis was supported (β = 0.430, *p* = 0.001), affirming that social influence substantially determines social media usage among healthcare workers. This corresponds with the Digital Engagement Theory, which argues that the actions of people are influenced by intrinsic motivations and external interactions [24]. In the healthcare environment, colleague norms and workplace culture govern appropriate digital participation, thus influencing users’ implementation trends. Previous research confirms this, noting that experts are inclined to interact in online tools when affected by esteemed associates and institutional standards [17]. The third hypothesis is accepted, with a beta coefficient of 0.430 and a significant *p*-value of 0.001. Thus, this confirms the vital role of social influence in determining medical workers’ engagement in social media. It has also been documented that prior research indicates that peer influence and institutional norms severely impact individuals’ adoption of digital technologies within professional settings. Since healthcare has a substantial degree of collaboration, professionals will follow how their colleagues behave and recommend using social media. This is why social influence is critical to digital brand activation.

**H4:**  *Information Sharing → Social Media Usage.*

The fourth hypothesis was significant (β = 0.579, *p* = 0.000), suggesting that healthcare workers who participate in information sharing are active on social media.

As the Professional Productivity Theory suggests, online platforms facilitate colleague communication, knowledge dissemination, and cooperative learning. This aligns with the results that highlight the benefits of healthcare dialog and effective communication in a healthcare environment where prompt access to knowledge can significantly influence decision-making and healthcare delivery. The fourth hypothesis (H4: information sharing → social media usage), with a beta coefficient value of 0.579 and a *p*-value of 0.000, is accepted, indicating that information-sharing behaviors significantly influence social media usage. According to prior research, social media is conducive to expediting knowledge transmission among workers, further increasing their effectiveness and decision-making [25]. This relationship is validated because information sharing is critical to improving practices and patient safety in the healthcare industry.

**H5:**  *Social Media Usage → Productivity.*

The final hypothesis assessed (H5) has been strongly accepted (β = 0.577, *p* = 0.000), showing that increased social media usage is associated with higher perceived productivity. This aligns with the Professional Productivity Theory, which argues that contextual platforms can facilitate productivity, and supports the notion that social media can improve productivity when properly utilized among healthcare workers [26]. The hypothesis (H5: productivity → social media usage) has been strongly accepted (β = 0.677, prob = 0.000), showing that social media raises productivity. In fulfilling existing studies, social media aids communication, collaboration, and knowledge sharing to improve work efficiency [27]. Accessing digital platforms in healthcare aids professionals by allowing them to obtain crucial information much quicker, thus improving productivity. The hypothesis testing results match the essential theories of adopting technology, digital engagement, and professional productivity. This underlines the significance of social media in influencing current healthcare practices and provides excellent insights as to how it can be leveraged to optimize digital strategies for improved performance.

### 5.4. Contribution

The study explores the dimension of social media on the productivity of healthcare workers. This study adds to the body of knowledge already available in a variety of healthcare professions and closes a research gap in the literature on health education in Nigeria [28]. For the government, this study helps stakeholders and policymakers understand the health information needs of students, aiding in the development of health policies that ensure better access to healthcare and quality treatment [29]. In summary, this study has contributed to online branding literature, as it shows conceptual and empirical evidence of the impact of the social media age on healthcare professionals.

### 5.5. Managerial Implications

The purpose of this study was to give better insight into social media dimensions and productivity among healthcare workers. The path analysis results obtained show that social media is used for information sharing and increases health productivity. The result is in line with a study [30] which revealed that social media can be a beneficial tool for educational purposes. Social media platforms enable medical practitioners to disseminate trustworthy, superior information and health messages that have a higher chance of connecting with and being embraced by patients. Hence, encouraging and promoting trust. By encouraging collaboration and facilitating the transmission of research, social media plays a critical role in healthcare, medical education, and research [31].

To verify and actively engage in the decision-making process, social media provides HCPs with online health information during consultations [32]. Social media is essential to modern healthcare because it makes it easier for experts to communicate with one another [33]. According to one study, social media can also encourage behavior change but estimating how much of this change will occur over time is outside the purview of social media health initiatives [34].

Furthermore, trust in social media platforms appeared as a significant component that influences productivity. This requires actions to foster confidence among healthcare workers in using social media tools by selecting reliable content and encouraging ethical guidelines. When trust is reinforced, healthcare workers can inspire meaningful engagement on social media platforms [35]. Healthcare organizations should create explicit policies and deliver training to guarantee that they align with the goals of the organization and improve employee productivity. The findings propose that variables such as information sharing and social influence have a measurable influence on productivity, emphasizing the benefits of promoting an environment that supports cooperation and knowledge sharing.

The path analysis results also show that social media networks can lead to FoMO. The fear of missing out and social influence has refuted necessary professional boundaries. According to the literature, social media interaction and boundary management are more challenging online than they are offline [36]. Additionally, social media distractions can result in serious mistakes, including prescription errors and poor communication during patient handoff [37]. The association between turnover intention and using social media for work outside of work hours is mediated by exhaustion. Using social media for business purposes outside work hours could potentially have unfavorable effects. People who use ICT excessively may experience a type of stress known as “technostress,” which can cause deficits [38]. Among its many manifestations, technology-induced stress is most noticeable when job obligations are transferred into personal time due to technology use [39].

### 5.6. Limitations and Further Research Directions

The study provided much information on social media but had limitations that should be considered. The study depended on the personal assessment of healthcare professionals; this could lead to biases in the data. Furthermore, not all facets of these intricate ideas may be captured by the productivity and social media use metrics used in the study. Hence, further studies need refinement for the constructs. The study had low factor loadings, which showed a weak indicator. This was retained because of their conceptual relevance and exploratory research adopted by the study. These drawbacks show how much more research is required to completely comprehend how social media affects healthcare productivity. Intervention research to assess measures to reduce social media distraction and enhance productivity among healthcare professionals should be conducted. Comparative research exploring social media dimensions and productivity among healthcare workers, as well as assessing other impacts aside from distraction and productivity. Studies should be conducted on the relationship between social media and the mental health of healthcare professionals.

## 6. Conclusions

This research offers both theoretical and evidence-based validation on social media dimensions and productivity among healthcare workers. The results indicate that although social media can be an effective tool in fostering trust, enabling professional interaction, and improving access to prompt medical knowledge, its overuse or unmanaged usage can lead to a lack of focus, reduced productivity, and weakened patient service. To address this, hospital administrators should enforce well-defined roles and guidelines for in-hospital procedures. This should be reinforced with regular professional development, training employees on appropriate engagement, dangers of overuse, and methods for coordinating online participation and healthcare obligations. The focus should be centered on utilizing tools for medical information and sharing data while minimizing irrelevant interruptions during office hours. Policymakers are urged to create legal structures that support morals as well as protect online involvement within healthcare settings. These structures should provide information safety, encourage evidence-based digital platforms, and support the implementation of nationally unified policies for social media use within professional care. For researchers, prospective studies should examine the long-term changes in social media interaction on healthcare productivity, evaluate how platform-based variations impact various medical roles, and assess improvement plans aimed at strengthening online knowledge among health workers. Enhanced focus is needed for cognitive and organizational dynamics such as fear of missing out (FoMO), trust in online tools, social influence, and information sharing that determine participation intensity and productivity results. This study is unique because most of the research on social media use is carried out in industrialized nations with high internet penetration rates. It offers a starting point for additional investigation into the social media phenomenon about health among Nigerian healthcare professionals.

## Figures and Tables

**Table 1:** Measurement model and reliability.

Construct	Factor Loading	Cronbach’s Alpha (α)	Composite Reliability	Average Variance Extracted (AVE)
SI	0.248	0.614	0.288	0.611
	0.361			
	0.266			
T	0.546	0.592	0.556	0.674
	0.421			
	0.422			
	0.559			
FoMO	0.742	0.584	0.826	0.641
	0.820			
	0.649			
	0.450			
	0.656			
	0.625			
IS	0.500	0.520	0.734	0.683
	0.554			
	0.688			
SMU	0.661	0.572	0.854	0.616
	0.623			
	0.661			
	0.688			
	0.797			
	0.661			
PI	0.596	0.622	0.693	0.645
	0.625			
	0.742			
	0.716			

**Table 2:** Discriminant validity.

	FOMO	IS	P	SI	SMU	T
FOMO	0.813					
IS	0.786	0.864				
P	0.680	0.703	0.825			
SI	0.700	0.609	0.596	0.872		
SMU	0.762	0.826	0.814	0.614	0.790	
T	0.803	0.651	0.616	0.814	0.666	0.833

**Table 3:** Summary of hypothesis test results.

Direct Effects		Beta	SE	t-Values	*p*-Values	Hypothesis
H1	(FOMO) → (SMU)	0.430	0.075	0.733	0.001	Accepted
H2	(T) → (SMU)	0.416	0.095	0.303	0.003	Accepted
H3	(SI) → (SMU)	0.430	0.075	0.733	0.001	Accepted
H4	(IS) → (SMU)	0.579	0.115	3.359	0.000	Accepted
H5	(SMU) → (P)	0.577	0.246	13.065	0.000	Accepted

**Table 4:** Structural model summary table.

Paths		β Coefficient	*p*-Values	f^2^ (Effect Sizes)	Result
H1	(FOMO) → (SMU)	0.430	0.001	0.097	supported
H2	(T) → (SMU)	0.416	0.003	0.055	supported
H3	(SI) → (SMU)	0.430	0.001	0.766	supported
H4	(IS) → (SMU)	0.579	0.000	0.010	supported
H5	(SMU) → (P)	0.577	0.000	0.061	supported

**Table 5:** Coefficient of determination (R^2^) for endogenous constructs.

Endogenous Construct	R^2^	Interpretation
Social Media Usage	0.663	0.001
Productivity	0.723	0.003

**Table 6:** Predictive relevance (Q^2^) for endogenous constructs.

Endogenous Construct	Q^2^	Interpretation
Social Media Usage	0.024	0.001
Productivity	0.129	0.003

## Data Availability

Data is contained within the article.

## Abbreviations

FOMO: Fear of Missing Out; IS: Information Sharing; SMU: Social Media Usage; SI: Social Influence; TR: Trust; PI: Productivity.

## Appendix A

**Table A1:** List of construct measurement items.

Construct	Items	Source
Social Media Usage	I often find myself using social media longer than intended.	[40]
	I often find life to be boring without social media.	
	Time passes by without me feeling it when I am using social media.	
	My work performance has deteriorated because of my social media usage. I find myself thinking about what happened on social media when I am away from it. I feel my social media usage has increased significantly since I started using it.	
Productivity	Social media helps me improve the quality of my work.	[41]
	Social media helps me accomplish more work than would otherwise be possible. Social media helps me to perform my job better.	
Information Sharing	It is acceptable if my personal health information is uploaded to social media.	[42]
	I am not bothered if my information is shared with another health institution connected to the social media platform.	
	Overemphasis on patients’ privacy protection hinders the necessary flow of information sharing among health institutions on social media.	
	Information sharing among the units and departments is effective.	
Fear of Missing Out	I fear my friends have more rewarding experiences than I do.	[40]
	I get anxious when I don’t know what my friends are up to.	
	I must understand my friend’s in-jokes.	
	It bothers me when I miss an opportunity to meet up with friends. When I have a good time, it is important for me to share the details online (e.g., updating my status). When I miss out on a planned get-together, it bothers me. When I go on vacation, I continue to keep tabs on what my friends are doing.	
Trust of the System	I trust social media to be reliable. I trust social media to be secure. I believe clinical interventions on social media are trustworthy.	[40]
	I trust clinical decision support systems on social media.	
